# The effect of synesthetic associations between the visual and auditory modalities on the Colavita effect

**DOI:** 10.1007/s00221-015-4363-0

**Published:** 2015-07-01

**Authors:** Jeroen J. Stekelenburg, Mirjam Keetels

**Affiliations:** Department of Cognitive Neuropsychology, Tilburg University, P.O. Box 90153, Warandelaan 2, 5000 LE Tilburg, The Netherlands

**Keywords:** Synesthetic congruency, Audiovisual integration, Colavita effect, Event-related potentials

## Abstract

The Colavita effect refers to the phenomenon that when confronted with an audiovisual stimulus, observers report more often to have perceived the visual than the auditory component. The Colavita effect depends on low-level stimulus factors such as spatial and temporal proximity between the unimodal signals. Here, we examined whether the Colavita effect is modulated by synesthetic congruency between visual size and auditory pitch. If the Colavita effect depends on synesthetic congruency, we expect a larger Colavita effect for synesthetically congruent size/pitch (large visual stimulus/low-pitched tone; small visual stimulus/high-pitched tone) than synesthetically incongruent (large visual stimulus/high-pitched tone; small visual stimulus/low-pitched tone) combinations. Participants had to identify stimulus type (visual, auditory or audiovisual). The study replicated the Colavita effect because participants reported more often the visual than auditory component of the audiovisual stimuli. Synesthetic congruency had, however, no effect on the magnitude of the Colavita effect. EEG recordings to congruent and incongruent audiovisual pairings showed a late frontal congruency effect at 400–550 ms and an occipitoparietal effect at 690–800 ms with neural sources in the anterior cingulate and premotor cortex for the 400- to 550-ms window and premotor cortex, inferior parietal lobule and the posterior middle temporal gyrus for the 690- to 800-ms window. The electrophysiological data show that synesthetic congruency was probably detected in a processing stage subsequent to the Colavita effect. We conclude that—in a modality detection task—the Colavita effect can be modulated by low-level structural factors but not by higher-order associations between auditory and visual inputs.

## Introduction

It is well established that for many multisensory events one sensory modality dominates the other. In the audiovisual domain, vision generally tends to dominate audition. Perhaps the most compelling demonstration of visual dominance is the Colavita effect, referring to the phenomenon that observers more often report the visual than auditory component of an audiovisual stimulus (Colavita 1974; Spence et al. 2012). Reversal of the Colavita effect, indicating auditory dominance, has almost never been reported in adults and was in fact only found in an n-1 repetition detection task (Ngo et al. 2011). Another well-known example of visual dominance is the ventriloquist effect, where the apparent sound location is attracted by a synchronously but spatially discordant visual stimulus (Welch and Warren 1980; Radeau 1994). The reverse, the effect of auditory location on the perception of visual location, is significantly less strong (Bertelson and Radeau 1981; Radeau and Bertelson 1987). Developmental studies have found auditory dominance at early age and show that visual dominance develops during childhood (Nava and Pavani 2013). Nava and Pavani (2013) found auditory dominance for the 6- to 7-year-old children, while adult-like visual dominance started to emerge reliably from 9 to 10 years of age.

Multisensory interactions that give rise to the visual dominance have been shown to depend on two sets of conditions or constraints. The first are structural factors, referring to inherent properties of the stimulus such as temporal and spatial contiguity between sensory inputs of different modalities (Calvert et al. 2004). Synchronicity is particularly critical for the interaction of multisensory inputs as a unified multisensory percept is more likely to be obtained when multisensory cues are in close temporal proximity (Meredith et al. 1987). In interactions in which space is task relevant or involving overt or covert attentional, spatial colocation also facilitates multisensory integration (for a review, see Spence 2013). A larger Colavita effect has, for example, been found when (audiovisual) AV stimuli were synchronous as opposed to asynchronous (Koppen and Spence 2007a), and when stimuli were presented at the same spatial location as opposed to different locations (Johnson and Shapiro 1989; Koppen and Spence 2007c). The magnitude of the ventriloquist effect also depends on structural factors as it declines with increasing spatial and temporal disparity between auditory and visual stimuli (Slutsky and Recanzone 2001; Lewald and Guski 2003; Wallace et al. 2004).

The second set of constraints that may affect multisensory interactions are cognitive factors, such as semantic, contextual or phonetic correspondences between the unimodal components, which provide a priori knowledge about the stimuli and how these are related. Informational audiovisual congruency, as found in naturalistic stimuli (de Gelder and Bertelson 2003), might help us to decide whether inputs from different senses belong to the same event (Bertelson 1999; de Gelder 2000; Dolan et al. 2001; Laurienti et al. 2004; Stekelenburg and Vroomen 2007; Noppeney et al. 2008). While it is generally acknowledged that structural factors are critical in audiovisual interactions at the behavioral and neural level, it is still debated whether cognitive factors such as semantic associations are equally important, or at what processing stage these factors might affect multisensory interactions. Laurienti et al. (2004), for example, showed that semantic associations between visual and auditory stimuli can facilitate multisensory stimulus processing because in a redundant cue feature discrimination task, task performance was better for semantically congruent than incongruent stimuli. Neuroimaging and electrophysiological studies also found semantic influences on the neural correlates of audiovisual integration (Molholm et al. 2004; Hein et al. 2007; Stekelenburg and Vroomen 2007; Yuval-Greenberg and Deouell 2007). Semantic congruency, however, had no effect on the ventriloquist effect (Radeau and Bertelson 1977, 1978). Furthermore, semantic congruency between pictures and sounds influenced the magnitude of the Colavita effect in a study of Stubblefield et al. (2013), but not in a study of Koppen et al. (2008).

Apart from semantic, contextual or phonetic correspondences between the individual components of multisensory events, associations between the senses can also be formed on the basis of natural (synesthetic) correspondences, referring to the phenomenon that observers tend to associate basic stimulus features (e.g., pitch, size or brightness) or dimensions of stimuli across sensory modalities (Spence 2011; Klapetek et al. 2012). As an example, observers typically associate a small-sized visual stimulus (e.g., a circle) with a high-pitched sound, and a large-sized visual stimulus with a low-pitched sound (Gallace and Spence 2006; Evans and Treisman 2010). Synesthetic audiovisual associations can be found in visual size, brightness, shape and auditory loudness, pitch and waveform shape, and are also found in other sensory modalities (Martino and Marks 2000), and are present early in infancy (Dolscheid et al. 2014; Walker et al. 2014). A number of studies report that synesthetic correspondences between different modalities affect cross-modal interactions. This has been demonstrated in cross-modal speeded classification paradigms in which the RT in response to synesthetically congruent stimuli is faster than to incongruent stimuli (Marks 1987; Gallace and Spence 2006; Evans and Treisman 2010). Furthermore, several studies show that synesthetic congruency actually modulates multisensory integration. In these studies, synesthetic congruency between visual size and auditory pitch affected the spatial ventriloquist effect (Parise and Spence 2009; Bien et al. 2012), and audiovisual temporal order judgment (TOJ) (Parise and Spence 2009). For the temporal ventriloquist effect, the findings are mixed. Whereas Parise and Spence (2008) found an effect of synesthetic congruency on the size of the temporal ventriloquist effect, Keetels and Vroomen (2007) report no such effect.

The majority of the studies thus suggest that multisensory synesthetic associations may strengthen the binding between the senses and therefore facilitate multisensory integration (see for a review Spence 2011). Here we examined whether synesthetic associations between visual and auditory stimuli affect the Colavita effect. The reason for expecting such an effect is that synesthetic associations between auditory and visual parts of the audiovisual event are thought to increase the unity assumption (i.e., the belief that two unimodal stimuli belong to the same sensory event). With increasing strength of the unity assumption, there is a higher chance that the visual stimulus adequately describes the AV stimulus, thereby eclipsing the auditory stimulus and making the auditory percept redundant (Koppen and Spence 2007a). It is, however, not self-evident that there will be an effect of synesthetic congruency because although the size of the Colavita effect is modulated by structural factors that are critical in multisensory integration, effects of semantic congruency on the Colavita effect are less consistent (Koppen et al. 2008; Stubblefield et al. 2013). However, if synesthetic congruency indeed increases multisensory binding, we expect that the magnitude of the Colavita effect will be larger for synesthetically congruent stimuli than for incongruent stimuli. We used similar audiovisual stimuli as in the aforementioned studies on size/pitch congruency because size/pitch congruency has shown to affect several types of audiovisual interactions. We also measured EEG to track the time course of synesthetic congruency. By contrasting event-related potentials (ERPs) of the synesthetic congruent and incongruent audiovisual stimuli, we examined at what stage of perception synesthetic congruency is processed (cf. Bien et al. 2012).

## Methods

### Participants

Twenty (13 women, mean age 21.1 years, SD 2.2) right-handed, healthy participants took part in the experiment. All were students from Tilburg University who reported normal hearing and normal or corrected-to-normal vision. All of them were naive to the purpose of the study. They received course credits for their participation. Written informed consent was obtained from all participants. This study was conducted in accordance with the Declaration of Helsinki.

### Stimuli and procedure

The experiment took place in a dimly lit and sound-attenuated room. Visual stimuli were presented on a 19-inch CRT monitor positioned at eye level, at 70 cm from the participant’s head. The sounds emanated from two speakers positioned on the left and right of the monitor. Stimuli were similar to those used in other studies on audiovisual synesthesia (Parise and Spence 2008, 2009; Bien et al. 2012). The visual stimulus was a 200-ms filled white circle on a black background with a diameter of either 1.3° (small) or 5.5° (large) of visual angle. The sounds were 200-ms pure tones of 300 Hz (low) or 4500 Hz (high), including 5-ms rise/fall times. Both sounds were played at 65 dB(A) and were perceived as equally loud. The visual stimuli were presented at the center of the screen. The auditory stimuli appeared as coming from the center location by presenting equally loud sounds from both speakers. There were eight different stimuli comprising auditory low, auditory high, visual small, visual large, auditory low/visual small, auditory low/visual large, auditory high/visual small and auditory high/visual large. In total, there were 500 trials for each unimodal condition and 250 trials for each bimodal condition, amounting to a total of 3000 trials. The eight conditions were presented in random order. The experiment was divided into 5 sessions of 600 trials. Each session was subdivided into 10 small blocks of 60 trials. Participants were allowed to have a mini-break after each 60 trials. Longer breaks were inserted between sessions. In each trial, one of the eight different stimuli was presented to which participants made speeded responses in order to report the stimulus category with either the index finger (auditory stimulus), middle finger (audiovisual stimulus) or ring finger (visual stimulus) of the right hand. Participants were instructed to respond as fast and accurately as possible. They were informed that sounds could be high or low and visual stimuli could be large or small, but it was also pointed out that this was irrelevant for the execution of the task. After the response, the next trial started after a random interval of 1500–2500 ms. The experiment was preceded by a short practice session of 24 trials.

### EEG recording and analysis

The EEG was recorded at a sample rate of 512 Hz from 64 locations using active Ag–AgCl electrodes (BioSemi, Amsterdam, The Netherlands) mounted in an elastic cap and two mastoid electrodes. The electrodes were placed according to the extended International 10–20 system. Horizontal and vertical eye movements were recorded using electrodes at the outer canthus of each eye and above and below the right eye, respectively. Two additional electrodes served as reference (Common Mode Sense active electrode) and ground (Driven Right Leg passive electrode). EEG was referenced offline to an average of left and right mastoids and band-pass-filtered (.1–30 Hz, 24 dB/octave). The 50-Hz interference was removed by a 50-Hz notch filter. The raw data were segmented into epochs of 900 ms, including a 100-ms prestimulus baseline. ERPs were time-locked to visual and auditory onset. After EOG correction (Gratton et al. 1983), epochs with an amplitude change exceeding ±150 μV at any EEG channel were rejected. The epochs of the congruent (auditory low/visual large; auditory high/visual small) and incongruent (auditory low/visual small; auditory high/visual large) stimuli were collapsed into two separate averages (congruent and incongruent).

## Results

### Behavioral results

**Fig. 1:**
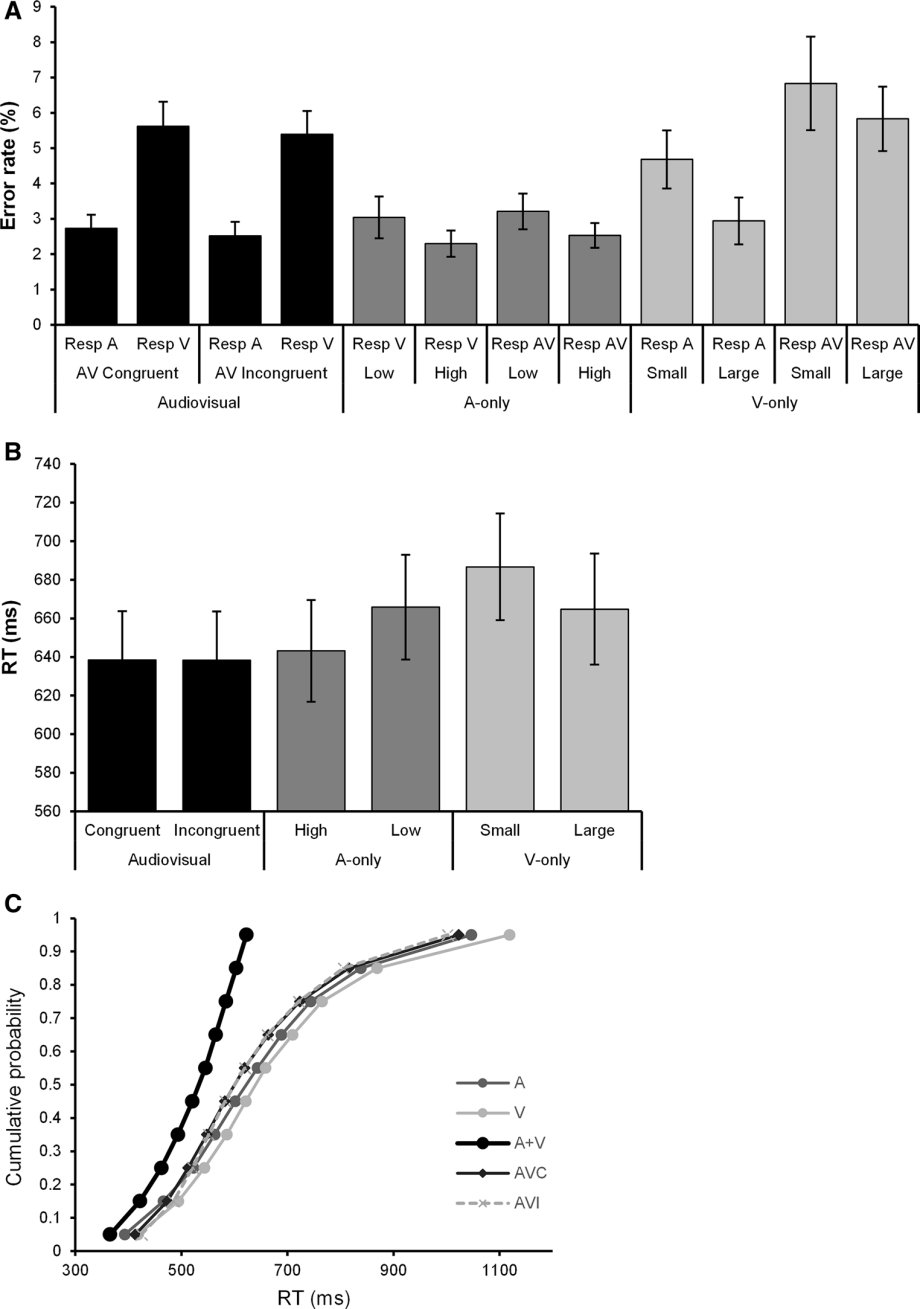
**a** The mean error rates for the unimodal and audiovisual stimuli. The unimodal conditions comprised auditory high and low pitch, visual small and large stimulus. The audiovisual conditions were either congruent (visual small/auditory high; visual large/auditory low) or incongruent (visual small/auditory low; visual large/auditory high). The erroneous responses are denoted by response (Resp) A (auditory), V (visual) and AV (audiovisual). **b** RTs for the unimodal and congruent and incongruent audiovisual stimuli. Error bars ± SEM. **c** Cumulative RT distribution functions (CDF) for the A, V, congruent (AVC), incongruent (AVI) conditions and the race model predictions (sum of the A and V CDFs: A + V)

In Fig. 1a, the error rates are displayed for the unimodal and audiovisual stimuli. As observed in other studies (Koppen and Spence 2007b; Koppen et al. 2008), more errors were made to visual-only stimuli (small stimulus: 4.7 % auditory and 6.8 % audiovisual responses; large stimulus: 2.9 % auditory and 5.8 % audiovisual responses) than to auditory-only stimuli (high-pitched tone: 2.3 % visual and 2.5 % audiovisual responses; low-pitched tone: 3.0 % visual and 3.2 % audiovisual responses). Figure 1a shows that for the audiovisual stimuli, more visual than auditory responses were given (i.e., the Colavita effect) and that audiovisual congruency had no effect on this response pattern. To test this latter observation more formally, we calculated the percentage of visual and auditory responses (error rates) for the audiovisual trials for both congruent (auditory low/visual large; auditory high/visual small) and incongruent (auditory low/visual small; auditory high/visual large) stimuli. We first ran tests of normality on the proportions: They were all nonsignificant. The audiovisual error scores were submitted to a repeated-measures ANOVA with the within-subject variables Response (A vs. V) and Congruency (congruent vs. incongruent). Indicative of the Colavita effect, there was a main effect of Response, *F*(1,19) = 26.49, *p* < .001, *η*_*p*_^2^ = .58, showing that when participants made errors in the audiovisual trials, they responded more with a visual response (5.5 %) than with an auditory response (2.6 %). There was no main of effect of Congruency, *F*(1,19) = 1.01, *p* = .327, *η*_*p*_^2^ = .05, and crucially no Response × Congruency interaction, (*F* < 1, *η*_*p*_^2^ < .001), indicating that synesthetic congruency had no effect on the magnitude of the Colavita effect. However, it should be noted that traditional null-hypothesis testing is unable to draw scientific conclusions from a statistically nonsignificant result. In contrast, Bayesian statistics can determine whether nonsignificant results support a null hypothesis over the alternative hypothesis, or whether the data are just insensitive (Wagenmakers 2007). We therefore conducted a Bayesian repeated-measures ANOVA (in JASP, https://jasp-stats.org/). The Bayes factor (BF_10_) for the main effect of Response, Congruency and the interaction between them was BF_10_ = 1.96e + 8, BF_10_ = .25 and BF_10_ = .28, respectively. When Bayes factor lies between .33 and 3, the data are insensitive; when the Bayes factor is larger than 3, the H1 is supported; and when the Bayes factor is smaller than .33, the H0 is supported (Raftery 1995). For the interaction, the Bayes factor indicates that the data are 3.6 (1/.28) times more likely under the null hypothesis (i.e., no effect of synesthetic congruency on the Colavita effect) than under the alternative hypothesis.

For the analysis of the reaction times (RTs), RTs of the auditory (high and low) and visual stimuli (small and large) were collapsed in a single average per modality. RTs of the congruent (auditory low/visual large; auditory high/visual small) and incongruent (auditory low/visual small; auditory high/visual large) stimuli were collapsed into two separate averages (congruent and incongruent). RTs differed between A, V, AVC (audiovisual congruent) and AVI (audiovisual incongruent) stimuli, *F*(3,17) = 5.89, *p* < .01, *η*_*p*_^2^ = .51, BF_10_ = 54.04 (Fig. 1b). Bonferroni-corrected post hoc tests revealed faster RTs for AVC and AVI stimuli compared to V (*p* values <.01). RTs to incongruent and congruent presentations did not significantly differ (*t* < 1, BF_10_ = .23). Next, we tested multisensory response enhancement separately for congruent and incongruent stimulus pairings by comparing the fastest unimodal RT with the bimodal RT: min(RT_A_, RT_V_) = RT_AV_. Responses for both congruent and incongruent stimuli were not faster compared to the fastest response to either of the unimodal stimuli: for both congruent and incongruent *t* values <1, and BF_10_ values =.33. We further evaluated the presence of multisensory response facilitation by implementing Miller’s test of the race model to determine whether response facilitation exceeded the statistical facilitation predicted by probability summation (Miller 1982). Per participant and separately for A, V, AVC and AVI, the cumulative distributions (CDF) of RT were estimated and the sum A and V CDFs (i.e., the race model, representing the upper bound of statistical facilitation) were computed. The race model inequality was tested at 10 percentile points (5th…95th) using Bonferroni-corrected t tests. Larger probabilities in the audiovisual conditions than the race model at any given percentile indicate integration. As depicted Fig. 1c for both congruent and incongruent pairings, there were no violations of the race model as the sum of the unimodal CDF (i.e., A + V in Fig. 1c) was larger than the CDF of either AVC and AVI. Furthermore, Fig. 1c shows that the CDFs for congruent and incongruent stimuli were practically identical. The analyses of RT thus show that in the current task, there was no redundancy gain for audiovisual stimuli over unimodal stimuli and AV congruency had no differential effect on the RT.

### ERP results

**Fig. 2:**
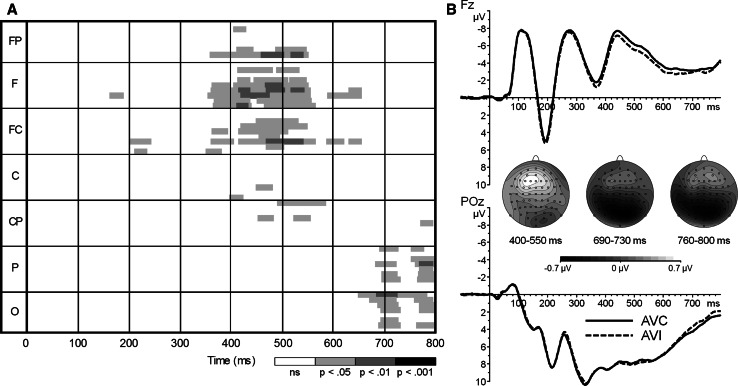
**a** Point-wise running *t* tests of the AV difference wave (incongruent–congruent) tested against prestimulus baseline activity in a 1- to 800-ms window at fronto-polar (FP), frontal (F), fronto-central (FC), central (C), centro-parietal (CP), parietal (P) and occipital (O) regions. The plot is highlighted only if at least 12 consecutive points were significant. **b** Comparison between congruent (visual small/auditory high; visual large/auditory low) and incongruent (visual small/auditory low; visual large/auditory high) grand averaged ERPs at electrodes Fz and POz. The scalp distribution of the AV difference wave (incongruent–congruent) plotted in 400–550 ms, 690–730 ms and 760–800 ms windows

First, an exploratory analysis of the spatio-temporal properties of AV congruency was conducted. The AV congruent ERP was subtracted from the AV incongruent ERP. The AVI–AVC difference wave was tested against prestimulus baseline levels by point-by-point two-tailed t tests at each electrode in a 1–800-ms window. Using a procedure to minimize type I errors (Guthrie and Buchwald 1991), differences between congruent and incongruent AV activities were considered significant when at least 12 consecutive points (i.e., ~23 ms) of the difference wave were significantly different from zero. This analysis allows for the exploration of the exact time course and location on the scalp where activity of incongruent AV presentations deviated from the congruent stimuli. Figure 2a shows that consistent differences in activity between the two congruency conditions are found at the frontal sites in a window of approximately 400–550 ms and subsequently at the occipitoparietal sites in a window of 690–730 ms and 760–800 ms. To test the difference in activity between the two congruency conditions across the 400- to 550-ms time window, the mean activity in a 400- to 550-ms window was calculated for both AV congruent and incongruent ERPs and submitted to a repeated-measures ANOVA with the within-subject variables Electrode (F3, F1, Fz) and Congruency (congruent vs. incongruent). There was an effect of Electrode *F*(2,18) = 22.52, *p* < .001, *η*_*p*_^2^ = .71, BF_10_ = 436.3, with the largest amplitude at Fz. There was a main effect of Congruency, *F*(1,19) = 10.93, *p* < .01, *η*_*p*_^2^ = .37, BF_10_ = 463.0, that did not depend on Electrode (*F* < 1, *η*_*p*_^2^ = .07, BF_10_ = .12). Mean activity for incongruent stimuli was .6 μV more positive than for congruent stimuli in the 400- to 550-ms time window (Fig. 2b). For activity in the 690- to 730-ms and 760- to 800-ms windows, there were Electrode × Congruency interactions, *F*(2,18) = 6.13, *p* < .01, *η*_*p*_^2^ = .41, BF_10_ = 9240.0, and *F*(2,18) = 3.30, *p* < .05, *η*_*p*_^2^ = .32, BF_10_ = 3.7, respectively. Simple effect tests of the interactions revealed that for both temporal windows, the activity was more negative for incongruent AV pairings (*p* values <.03, BF_10_ values >3.4, except for PO3 in the 760- to 800-ms window, BF_10_ = 2.0).

**Fig. 3:**
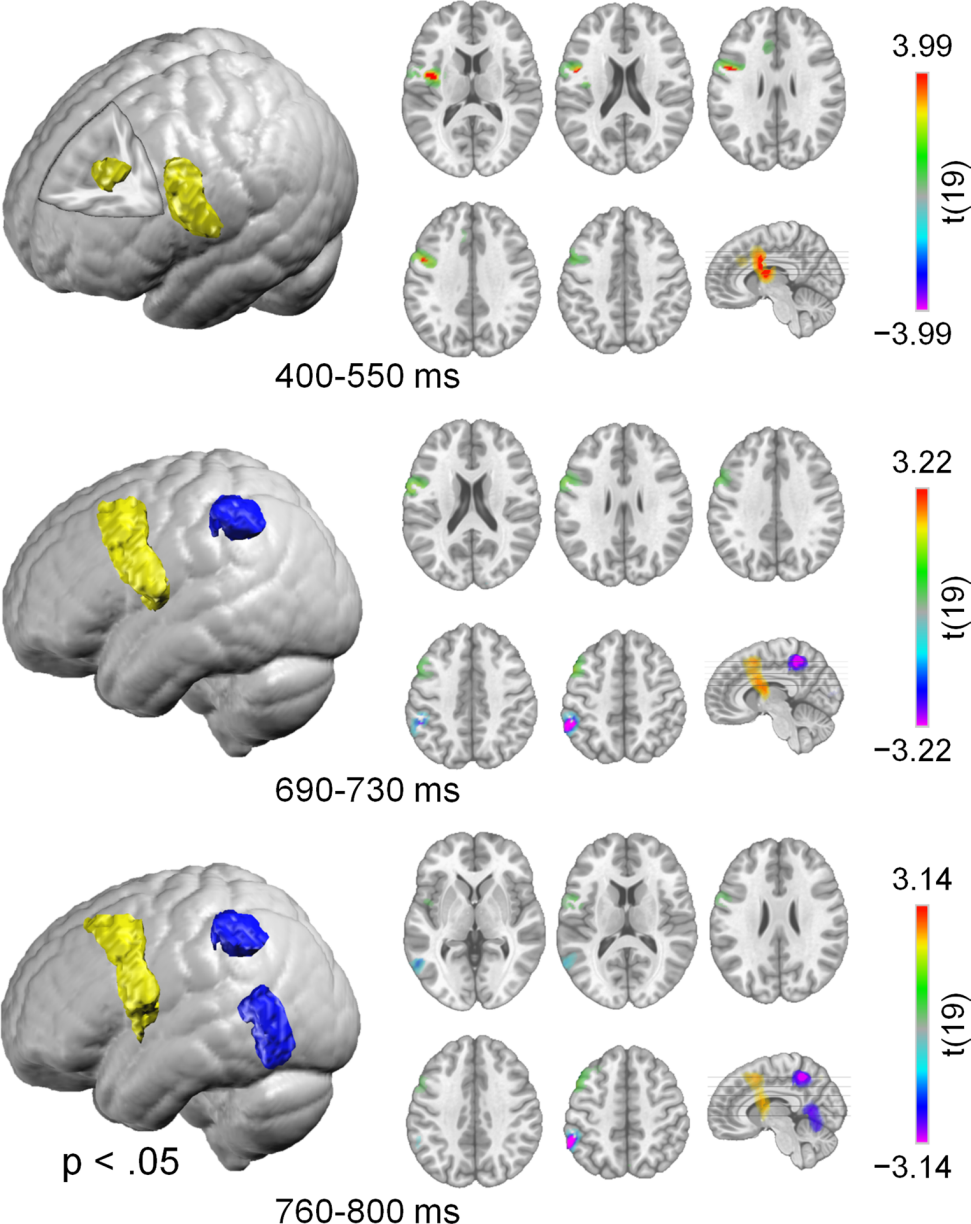
Statistical comparison of LAURA source estimation between the congruent and incongruent audiovisual presentations in 400–550 ms, 690–730 ms and 760–800 ms windows

We examined the neural sources underlying the difference in activity in the 400- to 550-ms, 690- to 730-ms and 760- to 800-ms windows associated with stimulus congruency using the LAURA (Local Auto-Regressive Average) distributed linear inverse solution (Grave de Peralta Menendez et al. 2001). LAURA estimates three-dimensional current density distributions calculated on a realistic head model with 5005 solution nodes equally distributed in the gray matter of the average MNI (Montreal Neurological Institute) brain. LAURA makes no a priori assumptions regarding the number of sources or their locations and can deal with multiple simultaneously active sources. This analysis was performed using the Cartool software by Denis Brunet (brainmapping.unige.ch/cartool). The LAURA inverse solution was estimated for each participant for both congruent and incongruent conditions in the three windows. To test the congruency effect, within-subject *t* tests were conducted on a node-by-node basis comparing the incongruent and the congruent estimated activity in source space. Because of the large number of t tests, correction for multiple tests has to be based on the number of independent measures. For EEG, this is the number of electrodes on the scalp, rather than the number of voxels (solution points) (Michel et al. 2004). Therefore, p values were corrected for the number of electrodes by the Bonferroni correction method. Only nodes where the differences were smaller than an alpha of .05 were reported. As shown in Fig. 3, activity associated with synesthetic congruency in the 400- to 550-ms window was localized in the anterior cingulate (ACC, BA 32/24) and the left precentral gyrus (BA 6). For the 690- to 730-ms and 760- to 800-ms windows, similar activity was found in the left precentral gyrus but not in ACC. Additional sources for the two late windows were the inferior parietal lobule (IPL, BA 40) and the posterior middle temporal gyrus (BA 21/BA 73) for the 760- to 800-ms window.

## Control experiment

The behavioral results show no evidence that synesthetic associations affect the Colavita effect. Although the stimuli were specifically chosen on the basis of previous studies that did show an effect of synesthetic congruency on audiovisual interactions with the same stimuli, it might be the case that the stimuli we used simply failed to establish a perception of synesthetic congruency (although we did find an effect of synesthetic congruency in the ERP). Alternatively, synesthetic congruency interactions might depend on the used task. To rule out the possibility that our stimuli were incapable of evoking a sense of synesthetic association, it would be reassuring if we could demonstrate audiovisual synesthetic interactions at the behavioral level with a speeded classification paradigm (Gallace and Spence 2006; Evans and Treisman 2010). Therefore, we tested 10 new right-handed participants (9 women, mean age 19.8 years, SD 1.6), who performed a 2AFC RT task in which either visual size or sound frequency was discriminated using two dedicated buttons. There were two separate blocks, one for the auditory and one for the visual target, each containing 100 congruent (auditory low/visual large; auditory high/visual small) and 100 incongruent (auditory low/visual small; auditory high/visual large) AV stimuli. Stimulus order was random, the intertrial interval varied between 1500 and 2500 ms, and the order of the two blocks was counterbalanced across participants. For the auditory target blocks, RT to the congruent AV stimuli was 19.8 ms faster than for incongruent AV stimuli, *t*(9) = 2,83 *p* < .05, *d* = .90, BF_10_ = 3.63. For the visual target blocks, RT to congruent AV stimuli was 11.8 ms faster than for AV incongruent stimuli, *t*(9) = 2,88 *p* < .05, *d* = .91, BF_10_ = 3.84. These results are in line with other studies using similar AV stimuli (Gallace and Spence 2006; Evans and Treisman 2010) and show cross-modal interference induced by the nontarget modality. The data thus show that synesthetic AV interactions can be achieved with the stimuli used in this study.

## Discussion

The current study replicated the Colavita visual dominance effect (Colavita 1974; Koppen and Spence 2007a, b, c; Koppen et al. 2008). When participants made an erroneous response in the audiovisual trials, they reported more frequently to have perceived a visual stimulus than an auditory stimulus. It was expected that synesthetic congruency would modulate the magnitude of the Colavita effect because synesthetic congruency has been shown to affect other manifestations of audiovisual integration (Parise and Spence 2008, 2009; Bien et al. 2012), while at the same time the Colavita effect is sensitive to (structural) factors that are critical for audiovisual integration (Koppen and Spence 2007a, c). We found, however, that the size of the Colavita effect was not affected by synesthetic congruency and that the Bayes factor favors the null hypothesis. In addition, RTs were unaffected by synesthetic congruency in the Colavita experiment, whereas in the speeded classification paradigm RT was higher for incongruent than for congruent audiovisual pairings.

It might be argued that the failure to find any effect of synesthetic congruency on the Colavita effect is due to participants simply not noticing that auditory and visual stimuli were coupled in different combinations (although they were informed about the pitch and size variations). Moreover, participants may not have experienced the audiovisual pairings as either congruent or incongruent. However, this argument is refuted by the control experiment showing a congruency effect in a speeded classification paradigm, which indicates that synesthetic associations between the auditory and visual modalities can be achieved with the currently used stimuli. Furthermore, the electrophysiological results showed that incongruent stimuli were processed differently than congruent stimuli because we found a late effect of synesthetic congruency in the ERP at approximately 400–550 ms at the frontal electrode sites and at 690–800 ms at the occipitoparietal electrodes. Others have also reported late audiovisual congruency effects, at 450 ms for semantically related stimuli (Molholm et al. 2004), at 300–500 ms for audiovisual speech (Klucharev et al. 2003; Lebib et al. 2004; Stekelenburg and Vroomen 2007; Baart et al. 2014), at 460–660 ms for speech–body actions (Meyer et al. 2013) and at 380–540 ms for letter–sound combinations (Raij et al. 2000). In some of these studies, the incongruent ERP was larger than the congruent ERP (Raij et al. 2000; Lebib et al. 2004; Molholm et al. 2004; Meyer et al. 2013), whereas in the current and in other studies it was the reverse (Klucharev et al. 2003; Stekelenburg and Vroomen 2007; Meyer et al. 2013; Baart et al. 2014). At the moment, we can only speculate about this difference in these congruency response patterns. It may be linked to the specific stimulus category or task or the interaction between them. For the current findings, however, the increased activity for congruent AV stimuli at the scalp and in ACC is fully in line with an fMRI study showing that semantically matching picture/sounds induced more activity in ACC than nonmatching stimuli (Laurienti et al. 2003). In the current study, congruent AV stimulation increased activity in the left precentral gyrus (BA 6) as well. Considering the fact that the activation is contralateral to the finger movement in the 400- to 550-ms window just before the response, it may be reasoned that it reflects premotor activation. If that were so, one might expect facilitation of RT in the congruent condition, which we did not find. To further examine whether the activity in BA 6 in the 400- to 550-ms window is motor related, we compared the neural source of the fast and slow AV trials based on the median split of RT. One would expect stronger activity in BA 6 for fast RTs than for slow RTs. However, we found no such difference, suggesting that the activity in BA 6 is not motor related. Alternatively, activity in the premotor cortex might be associated with the processing of synesthetic AV congruency. This would be in accordance with studies showing that congruency between audiovisual stimuli (biological motion) affected activity in the premotor cortex (Petrini et al. 2011; Wuerger et al. 2012). The synesthetic AV congruency interactions in the ACC and premotor cortex were followed by interactions in the IPL and pMTG. Both IPL and pMTG are multisensory regions that are sensitive to the congruency between auditory and visual stimuli (Jones and Callan 2003; Beauchamp et al. 2004; Taylor et al. 2006; Szycik et al. 2009). The time course and estimated sources underling synesthetic AV congruency indicate that in the current study, audiovisual congruency was processed at a late stage of stimulus processing.

A potential limitation of our interpretation of the late ERP effects is that these occurred without any congruency effect at the behavioral level. It should be noted though that dissociation between effects at the neural and behavioral levels is not uncommon. An fMRI study of Taylor et al. (2006) for example reports audiovisual semantic congruency effects at the neural level but not at the behavioral level. This does not necessarily imply that the manipulation of synesthetic congruency was ineffective but demonstrates that brain responses are often more sensitive to experimental manipulations than to behavioral measures (Wilkinson and Halligan 2004).

The speeded classification task in our control experiment confirmed audiovisual pitch-size response-compatibility effects of other studies (Gallace and Spence 2006; Evans and Treisman 2010). Although these findings show cross-modal synesthetic interference, interactions in a speeded classification task can occur at any level between sensory registration and decision/response selection (Evans and Treisman 2010; Klapetek et al. 2012). The task that induces the Colavita effect and other tasks measuring audiovisual integration (Keetels and Vroomen 2007; Parise and Spence 2008, 2009; Bien et al. 2012; Klapetek et al. 2012) minimize the influence of decision/response selection. Most of these studies report an effect of synesthetic congruency on different instances of audiovisual interactions, suggesting that these cross-modally congruent stimuli are integrated at a perceptual level. The question then is why there was no effect of synesthetic congruency on the Colavita effect. One reason might be that the synesthetic congruency effect on audiovisual interactions may depend on the nature of the task and/or task instruction. In two studies reporting synesthetic congruency effects on audiovisual integration, stimulus dimensions of both stimuli had to be explicitly compared between modalities (Parise and Spence 2009; Bien et al. 2012). Therefore, both unimodal stimuli were task relevant and under attentional focus. Active processing of both auditory and visual stimuli may have shifted the detection of audiovisual congruency forward in time compared to our study (cf. prior entry effect, see for review Spence and Parise 2010). This was observed in the study of Bien et al. (2012) who found that synesthetic congruency modulated the ventriloquist effect. In their study, the detection of synesthetic congruency occurred at approximately 250 ms in the ERP, which is about 150 ms earlier than in our experiment. Accordingly, as congruency is detected in an earlier stage, top-down influences can penetrate the processing stage at which the ventriloquist effect occurs: the mid-latency components of the ERP at about 200–260 ms (Stekelenburg et al. 2004; Bonath et al. 2007). In our study, auditory and visual stimuli were not directly compared and no effect of congruency on the Colavita effect was found. It might be that audiovisual synesthetic congruency modulates audiovisual integration primarily when the stimuli in both modalities are task relevant. Further support for this notion comes from a study showing that information about synesthetic congruency needs to be processed consciously or deliberately in order to have an effect on audiovisual interactions (Klapetek et al. 2012). Klapetek et al. (2012) investigated whether synesthetic congruency between the pitch of a cue sound and the brightness of the target modulated the pip-and-pop effect. The pip-and-pop effect refers to the phenomenon that the detection of a visual target among visual distractors is speeded up by a spatially uninformative auditory cue (Van der Burg et al. 2008). Cue-target congruency affected the pip-and-pop effect only when participants were explicitly informed about the pitch-brightness mapping and were encouraged to make use of this information. The results of Klapetek et al. (2012) show that synesthetic associations affect audiovisual integration when both visual and auditory features are actively attended to and are task relevant. Klapetek et al. (2012) argued that in their study most of the congruency effects occurred at the postperceptual stage of stimulus processing. This finding and the currently reported late congruency effects in the ERP may well account for the null finding at the behavioral level because audiovisual congruency is presumably detected at the processing stage subsequent to the level at which the Colavita effect occurs. This was also hypothesized by Koppen et al. (2008) who found no effect of *semantic* audiovisual congruency on the Colavita effect. Our study that used the same task as Koppen et al. (2008) is an extension of their observations for *synesthetic* congruency. It should be noted though that when participants were required to detect an amodal target concept (e.g., “cat”) (Stubblefield et al. 2013) instead of stimulus modality (Koppen et al. 2008) semantic audiovisual congruency did affect the Colavita effect. In the study of Stubblefield et al. (2013), the visual dominance effect was demonstrated by the finding that sound targets were missed significantly more often when presented together with a visual distractor than for semantically congruent audiovisual presentations. According to Stubblefield et al. (2013), visual input was more salient than auditory input because visual representations were rated as being more representative of a semantic concept than auditory representations. Therefore, for incongruent trials, participants respond more readily to visual stimuli than auditory stimuli when searching for a conceptual target. The reason why Stubblefield et al. (2013) but not Koppen et al. (2008) found an effect of synesthetic congruency on the Colavita effect may lie in the fact that the task in Stubblefield et al. (2013) study tapped into the processing of the stimuli at the semantic level, whereas the Koppen et al. (2008) study did not. This explanation would be in line with the finding that RTs for semantic incongruent stimuli are slower for incongruent audiovisual stimuli than for congruent stimuli when participants evaluated the semantic congruency between auditory and visual stimuli, whereas in a stimulus detection task, semantic congruency had no effect on RT (Diaconescu et al. 2011). The difference between the studies of Stubblefield et al. (2013) and Koppen et al. (2008) also corroborates the notion that task factors are important in the influence of higher-order associations on the Colavita effect. Based on the available data, we hypothesize that—when using the modality detection task—the Colavita effect is sensitive to bottom-up stimulus factors as the magnitude of the Colavita effect is affected by spatial and temporal coincidence between audio and visual signals (Koppen and Spence 2007a, c), but not to higher-order stimulus associations (either semantic or synesthetic), which are processed at the late processing stages.

To conclude, the present study did not find any influence of synesthetic correspondence between the size of visual stimuli and the pitch of auditory stimuli on the Colavita effect. Audiovisual synesthetic associations were probably processed in a stage subsequent to the stage in which the Colavita effect occurs. Earlier reports showing that synesthetic congruency modulates audiovisual interactions therefore do not generalize to the Colavita effect. Our findings suggest that the effect of synesthetic associations on audiovisual interactions may depend on the interplay between stimulus and task factors.
